# Acceptability of treadmill perturbation-based balance training in older adults at risk of falling: A mixed-methods evaluation of participant and trainer perspectives

**DOI:** 10.1007/s40520-025-03320-0

**Published:** 2026-01-16

**Authors:** Natalie Hezel, Theresa Buchner, Clemens Becker, Jürgen M. Bauer, Simon Steib, Christian Werner

**Affiliations:** 1 Geriatric Center, Medical Faculty Heidelberg, Heidelberg University, Heidelberg, Germany; 2 Department of Human Movement, Training and Active Aging, Institute of Sports and Sports Sciences, Heidelberg University, Heidelberg, Germany; 3 Network Aging Research Heidelberg, Heidelberg University, Heidelberg, Germany

**Keywords:** Acceptability of health care, Reactive balance, Health care professionals, Fall prevention, Implementation, Qualitative research

## Abstract

**Background:**

Perturbation-based balance training (PBT) specifically targets fall mechanisms and holds promise for fall prevention in older adults, but its reliance on near-fall exposure may pose a barrier to engagement. Successful implementation depends on acceptability among participants and trainers, yet a mixed-methods, multi-perspective evaluation of PBT acceptability is lacking.

**Aims:**

To evaluate the acceptability of treadmill PBT in older adults at risk of falling and in trainers, and to examine associations with participant characteristics.

**Methods:**

Twenty-nine participants (79.9 ± 5.5 years) completed a 6-week treadmill PBT intervention, delivered by three trainers. Retrospective acceptability was assessed using a questionnaire (maximum score: 35 pt. for participants, 30 pt. for trainers) and semi-structured focus groups (12 participants, all trainers), guided by Theoretical Framework of Acceptability (TFA) domains and additional context-specific topics. Associations between participant characteristics and questionnaire scores were analyzed using multivariate regression. Focus-group data were analyzed deductively using the TFA.

**Results:**

Median questionnaire scores were high for participants (28 [interquartile range, IQR 23–32] pt.) and trainers (26 [IQR 25–26] pt.). Fall history emerged as the only independent predictor of lower acceptability among participants. Focus groups revealed that both participants and trainers generally perceived PBT as acceptable. High perceived safety and effectiveness for improving reactive balance, adequate tailoring and supervision, and strong coherence were reported as facilitators. Potential barriers included anxiety, fall-related memories, the demanding nature of PBT, and setting-related factors (e.g., monotony, limited social interaction, missing handrails, narrow belt).

**Conclusions:**

Treadmill PBT were generally well accepted by trainers and older adults at risk of falling but showed lower acceptability among participants with fall history. Implementing PBT in individuals with no fall history may help mitigate anxiety related to prior fall experiences and support higher acceptability.

**Trial registration:**

DRKS00030805 (December 14, 2022).

**Supplementary Information:**

The online version contains supplementary material available at 10.1007/s40520-025-03320-0.

## Introduction

Falls among older adults pose a significant public health concern [1]. Physical exercise is one of the most evidence-based fall prevention strategies, with programs including balance, strength, and functional exercises reducing fall rates by about 25% when performed consistently (i.e., ≥ 3 sessions per week for ≥ 12 weeks) [2]. Perturbation-based balance training (PBT), which challenges reactive balance to regain postural stability after externally applied disturbances (e.g., slips, trips), has shown even greater promise, with studies suggesting fall rate reductions of up to 50% at lower training volume (e.g., 1–8 PBT sessions; 30–60 min per session) [3–5]. PBT can be implemented via different methods, including overground walkways with pop-up obstacles or low-friction movable platforms, manually applied perturbations by a trainer or device (e.g., lean-and-release or tether-release), or specialized treadmills that provide repeatable perturbations through belt accelerations or decelerations and/or lateral platform displacements at varying intensities [6]. The task-specific nature of PBT may explain its efficacy, as it directly targets slips and trips, among the leading causes of falls in older adults [7]. However, PBT at the limit of stability, mimicking near-fall situations [6], has been associated with tension and anxiety of participants [8–10]. This highlights the need for research to mitigate these side effects and ensure acceptability [6], as successful implementation of healthcare interventions relies on their acceptability to both participants and trainers [11].

Intervention acceptability has been proposed to enhance adherence and benefits for participants, and to support fidelity of delivery for trainers, both of which influence overall effectiveness [12, 13]. Sekhon et al. [11] developed the Theoretical Framework of Acceptability (TFA), which conceptualizes acceptability as a multi-faceted construct across seven domains (affective attitude, burden, perceived effectiveness, intervention coherence, ethicality, opportunity costs, self-efficacy), providing a systematic approach to evaluating the acceptability of healthcare interventions. While acceptability of PBT has previously been assessed using indirect measures such as satisfaction, side effects, or dropout rates [10, 11, 14], more recent studies have used qualitative semi-structured interviews [8, 15] or quantitative surveys [9], informed by the TFA. Semi-structured interviews with community-dwelling older adults with a recent history of falls [8] or with independently living older adults and healthcare professionals delivering PBT [15] indicated that PBT executed on a treadmill was generally perceived as acceptable, safe, and effective. A quantitative survey comparing such treadmill PBT with dynamic stability training in the presence of perturbations found both paradigms to be well accepted with no differences between them among fall-prone community-dwelling older adults; however, treadmill PBT, appeared more appropriate for anxious participants [9]. To date, a mixed-methods evaluation of PBT acceptability integrating both participant and trainer perspectives has not been conducted. Combining quantitative measures with in-depth qualitative insights into experiences, motivations, and concerns [16, 17] offers a more comprehensive and nuanced understanding than either method alone, yet such approaches are still underdeveloped in health disciplines [18–20].

To address this gap for PBT, this study conducts a mixed-methods evaluation of treadmill PBT acceptability in older adults at risk of falling and in trainers. The aim is to systematically assess retrospective acceptability among both participants and trainers via a TFA-guided questionnaire and focus groups, and to evaluate whether participant acceptability is associated with sociodemographic, clinical, cognitive, psychological and physical characteristics that may influence perceived acceptability. Such insights are crucial for optimizing PBT implementation and ensuring feasibility and sustainability in target groups.

## Methods

### Study design

This study used a convergent mixed-methods design and builds on data from the previously published two-arm randomized controlled FEATURE pilot trial, details of which are published elsewhere [21, 22]. In brief, the FEATURE trial examined the feasibility, acceptability, and dose-response relationship of treadmill PBT for improving reactive balance in older adults at risk of falling. While overall participant acceptability, assessed using the TFA questionnaire, was reported as high with no between-group differences [22], the present study provides a comprehensive evaluation of acceptability by integrating quantitative and qualitative data from participants and trainers, including analyses of individual TFA domains, and exploring factors associated with participant acceptability.

A quantitative survey and qualitative semi-structured focus groups were conducted and analyzed independently to assess the retrospective acceptability of treadmill PBT from the perspectives of participants and trainers. Integration of quantitative and qualitative data occurred during the interpretation phase. Reporting follows the GRAMMS (Good Reporting of A Mixed Methods Study) guidelines (Supplementary Table 1) [23]. The qualitative component was further informed by the COREQ (Consolidated Criteria for Reporting Qualitative Research) checklist (Supplementary Table 2) [24].

## Participants and context

Participants of the FEATURE trial were enrolled after completing the PBT intervention [21], originally consecutively recruited from a senior fitness club (REGE e.V.) associated with a German geriatric hospital (Agaplesion Bethanien Hospital Heidelberg). Inclusion criteria were age ≥ 65 years, being at risk of falling (Timed Up and Go [TUG] > 12 s [25], habitual gait speed < 1.0 m/s, and/or ≥ 1 fall in past 12 months), and ability to walk ≥ 2 min without walking aid. Exclusion criteria were cognitive impairment (Mini-Mental State Examination [MMSE] < 24 pt. [26]) and severe neurological, cardiovascular, metabolic, or psychiatric disorders.

Three sports science students trained to deliver PBT served as trainers in the FEATURE trial.

The two arms of the FEATURE trial consisted of six PBT sessions (6PBT) or two PBT sessions combined with four conventional treadmill sessions (2PBT), delivered once weekly over 6 weeks as part of the participants’ 90-min REGE e.V. group-based training session. The intervention was conducted between February to September 2023. Both arms are described in detail elsewhere [21]. Briefly, PBT sessions consisted of 40 unannounced, randomized anterior-posterior and mediolateral surface perturbations on a specialized treadmill (BalanceTutor™, MediTouch, Netanya, Israel), delivered in five blocks (1.5–3 min each) of eight. Perturbation magnitudes were individually adjusted and progressed based on combined participant and trainer ratings of perceived difficulty and anxiety, using 5-point Likert scales, targeting challenging but manageable levels [10]. Participants were secured with an overhead harness to prevent falls and underwent a full-body suspension before each session to familiarize them with the safety procedure. Conventional treadmill sessions consisted of five 3-min blocks of unperturbed walking on a standard medical treadmill (pluto med, h/p/cosmos sports & medical gmbh, Nussdorf-Traunstein, Germany).

## Acceptability measures

### TFA questionnaire

After the final PBT session, PBT acceptability was assessed using a 7-item TFA questionnaire, covering all seven TFA domains in the context of PBT (Supplementary Table 3). Each item was rated on a 5-point Likert scale, yielding a total score of 7–35 points, with higher scores indicating higher acceptability. At the end of the intervention period, trainers completed a 6-item version of the TFA questionnaire, excluding the opportunity costs domain (total score 6–30 pt.; Supplementary Table 3). Scores were categorized for participants as very high (30–35 pt.), high (25–29 pt.), moderate (19–24 pt.), low (14–18 pt.) and very low (7–13 pt.), and for trainers as very high (26–30 pt.), high (21–25 pt.), moderate (16–20 pt.), low (11–15 pt.) and very low (6–10 pt.).

## Focus groups

All participants and trainers were invited to take part in the focus groups and, if they were interested, approached via telephone by the research team. For participants, these were planned in small groups (3–5 persons) after completion of their intervention, with two sessions held per arm (June and September 2023) to accommodate staggered intervention completions and reduce recall bias. These focus groups included three or four participants, except for one group with two participants due to illness-related non-attendance at short notice. One focus group was conducted for all three trainers after 20 participants had completed the intervention (July 2023). Given the limited number of trainers, this focus group was conducted to obtain exploratory insights into acceptability, rather than to achieve thematic saturation or support generalizable conclusions. Each focus group lasted approximately one hour and was held at the study center (Agaplesion Bethanien Hospital Heidelberg).

Semi-structured focus groups were conducted in accordance with the TFA, with each of the seven domains translated into related questions [8, 15] to inform an interview guide (Table 1). Additional context-specific questions addressed PBT-related topics (training setting, tailoring, anxiety, and safety) identified in previous studies on PBT acceptability in older adults [8–10]. The discussion started with an introductory question (“What is the first thing that comes to your mind when you think of PBT?“), followed by the TFA-informed and context-specific questions, with additional follow-up prompts as appropriate.

Focus groups were primarily moderated by NH, who was not involved in the PBT, and attended by a second researcher (TB), who took field notes to support triangulation. All sessions were audio-recorded via smartphone application, transcribed verbatim, anonymized by removing names and other identifying information from transcripts, and all quoted material was carefully translated from German into English. All focus groups were completed prior to the primary analysis of the dose-response relationship of PBT on reactive balance.

**Table 1:** Focus group themes and guiding questions (according to Gerards et al. [8])

Theme	Guiding questions	
	Participants	Trainers
**TFA questions**		
Affective attitude – *“how an individual feels about the intervention”*	How do you feel about PBT? What made you feel this way?	How do you feel about (delivering) PBT? What made you feel this way?
Burden *–* *“the perceived amount of effort that is required to participate in or deliver the intervention”*	To what extent did you find PBT strenuous? To what extent did you find PBT challenging?	To what extent did you find delivering PBT strenuous? To what extent did you find delivering PBT challenging?
Ethicality *–* *“the extent to which the intervention has good fit within an individual’s value system”*	To what extent did PBT fit with your expectations of fall prevention?	To what extent did PBT fit with your expectations on fall prevention?
Intervention coherence *–* *“the extent to which the participant understands the intervention and how it works”*	In your own words, what was the aim of PBT?	In your own words, what was the aim of PBT?
Perceived effectiveness *–* *“the extent to which the intervention is perceived as likely to achieve its purpose”*	To what extent did you experience effects from PBT?	To what extent did you experience participants’ effects from PBT?
Self-efficacy *–* *“the participants confidence that they can perform the behavior(s) required to participate in the intervention”*	How did you do during PBT?	How did you do when delivering PBT?
Opportunity costs *–* *“the extent to which benefits*,* profits or values must be given up to engage in the intervention”*	To what extent did you forego other activities to participate in PBT?	- (not addressed as all trainers were part of the study team)
**Context-specific questions**		
Training setting	What are your thoughts about … … the duration of PBT/the number of PBT sessions? … the way PBT was supervised? … PBT individually as opposed to in a group? … the treadmill used in PBT?	
Tailoring	What are your thoughts about … … the way the PBT was tailored/progressed? … the scoring scales that were used during PBT?	
Anxiety and safety	Were you afraid (of falling) during PBT? Did you feel safe during PBT?	Were you afraid when delivering PBT? Did you feel safe when delivering PBT?

TFA = Theoretical Framework of Acceptability; PBT = perturbation-based balance training

### Descriptive measures

Age, sex, years of education, treadmill experience, fall history in the last 12 months, and Fried frailty phenotype [27] were assessed as sociodemographic and clinical characteristics. Cognitive functioning was evaluated using the MMSE [26] and Trail Making Test B-A [28], and concerns about falling using the Short Falls Efficacy Scale-International (Short FES-I) [29]. Physical capacity was measured with the TUG [25] and Short Physical Performance Battery [30]; gait capacity with the 4-m gait speed and 2-min walk tests [31]; global balance with the Brief-Balance Evaluation Systems Test [32]; dynamic balance with the Four Square Step Test [33]; and reactive balance with the Stepping Threshold Test [34, 35] and Dynamic Stepping Threshold Test [21, 22]. All descriptive measures were collected at baseline in the FEATURE trial. More details on the descriptive measures are provided in the Supplementary Material.

## Statistical and analytical procedures

Group differences were analyzed using Fisher’s exact, Mann-Whitney *U*, or independent-samples *t*-tests. Point-biserial (*r*_*pb*_), Spearman rank (*r*_*s*_) and Pearson (*r*_*p*_) correlation coefficients were calculated to examine bivariate associations between participant characteristics and the total TFA questionnaire score. Correlations were interpreted as low (< 0.30), moderate (0.30–0.50), or high (> 0.50) [36]. Participant characteristics that showed significant bivariate associations were entered into a linear multivariable regression model (enter method) to identify independent predictors of total TFA questionnaire scores. Statistical analyses were performed using IBM SPSS version 29.0 (IBM Corp., Armonk, NY, USA). Statistical significance was set at *p* <0.05.

Qualitative data were analyzed following a deductive approach [37] guided by the TFA domains [11] and other pre-identified PBT-related topics [8–10], performed by NH. Findings are presented along these domains and topics and illustrated by representative quotes from participants and trainers.

## Results

### Participant and trainer characteristics

Twenty-nine of 36 participants initially randomized in the FEATURE trial completed the TFA questionnaire after the final PBT session, and 12 additionally participated in the focus groups. Seven participants dropped out: four withdrew after the baseline reactive balance assessment due to anxiety about receiving further perturbations, and three withdrew during the intervention due to medical events unrelated to the PBT. Twenty-seven attended all PBT sessions, while two discontinued after the third or fourth training session due to hip pain or anxiety about the PBT. Detailed characteristics of the 29 participants (mean age = 79.9 ± 5.5 years, female: *n* = 19, 65.5%) who completed the quantitative survey are provided in Table 2.

All three trainers were female aged 21–23 years.

**Table 2:** Participant characteristics

Characteristic	Participants (*n* = 29)
Age, years	79.9 ± 5.5
Female, *n*	19 (65.5)
Treadmill experience, *n*	14 (48.3)
Education, years	13.7 ± 2.9
Fall history, *n*	12 (41.4)
Fried frailty phenotype (categories), *n*	
Robust	15 (51.7)
Pre-frail	11 (37.9)
Frail	3 (10.3)
Mini-Mental State Examination, pt.	28.3 ± 1.5
Trail Making Test B-A, s	85.6 ± 52.7
Short Falls Efficacy Scale-International, pt.	9 [8–11]
Timed Up and Go, s	12.5 ± 5.6
Short Physical Performance Battery, pt.	9.9 ± 2.8
4-m gait speed test, m/s	0.84 ± 0.18
2-min walk test, m	122.2 ± 34.9
Brief-Balance Evaluation Systems Test, pt. (*n* = 27)	12.3 ± 5.4
Four Square Step Test, s	13.7 ± 8.1
Stepping Threshold Test (ACE), pt. (*n* = 28)	17.0 ± 5.0
Stepping Threshold Test (DSE), pt. (*n* = 28)	21.0 ± 5.7

Descriptive data given as mean ± standard deviation, median [interquartile range], or *n* (%). ACE = all-step count evaluation, DSE = direction-sensitive evaluation

### TFA questionnaire

A high total TFA questionnaire score was observed for participants (median = 28 [interquartile range, IQR 23-32] pt.), Table 3). Two-thirds (*n* = 20, 68.9%) rated PBT as highly to very highly acceptable (≥ 25pt.). Only one participant (3.4%) rated it very low (≤ 13pt.). Six of seven domains had median scores ≥ 4 points; only burden was rated lower (median *=* 3 [IQR 1–5] pt.).

Trainer reported a similar high total score (median = 26 [IQR 25–26] pt.), with all rating PBT as highly to very highly acceptable (≥ 25pt.). Burden received also the lowest rating (median = 3 [IQR 3.0–3.5] pt.).

**Table 3:** TFA questionnaire scores of participants and trainers

Variable	Participants (*n* = 29)	Trainer (*n* = 3)
Domain scores, pt.		
Affective attitude	4.0 [2.0–5.0]	4.0 [3.5–4.5]
Burden	3.0 [1.0–5.0]	3.0 [3.0–3.5]
Ethicality	4.0 [4.0–5.0]	4.0 [4.0–4.5]
Intervention coherence	5.0 [4.0–5.0]	4.0 [4.0–4.5]
Perceived effectiveness	5.0 [4.0–5.0]	5.0 [4.5–5.0]
Self-efficacy	5.0 [3.0–5.0]	5.0 [4.5–5.0]
Opportunity costs	5.0 [3.0–5.0]	-
Total score, pt.	28 [23–32]	26 [25–26] ^a^
Categories, *n*		
Very high	11 (37.9)	2 (66.7)
High	9 (31.0)	1 (33.3)
Moderate	8 (27.6)	0 (0.0)
Low	0 (0.0)	0 (0.0)
Very low	1 (3.4)	0 (0.0)

Data given as median [interquartile range] or *n* (%). Total TFA questionnaire score for participants (max. 35 pt.) were categorized as very high = 30–35 pt., high = 25–29 pt., moderate = 19–24 pt., low = 14–18 pt., and very low = 7–13 pt. ^a^Total TFA questionnaire score for trainers (max. 30 pt.) were categorized as very high = 26–30 pt., high = 21–25 pt., moderate = 16–20 pt., low = 11–15 pt., and very low = 6–10 pt

### Associations between participant characteristics and total TFA questionnaire score

Moderate, significant negative correlations were found between the total TFA questionnaire score and fall history (*r*_*pb*_=−0.43, *p* =0.021) and Short FES-I (*r*_*s*_=−0.48, *p* =0.009; Table 4). No other variables correlated significantly, including the number of PBT sessions attended (*p* =0.051-0.980). In the multivariate regression, fall history emerged as an independent predictor of lower PBT acceptability (*β*_*std*_=−0.38, 95% confidence interval [CI] −0.71 to −0.02; *p* =0.038), whereas Short FES-I was not significant (*β*_*std*_=−0.28, 95% CI −0.61 to 0.07; *p* =0.116).

Median total TFA questionnaire scores were 24.5 [IQR 22.5–28.5] points in fallers and 30 [IQR 27–33] points in non-fallers.

**Table 4:** Bivariate and multivariable associations of participant characteristics with the total TFA questionnaire score

Participant characteristic	Bivariate analysis		Multivariable analysis^1^	
	*r* (95% CI)	*p*	*β*_*std*_ (95% CI)	*p*
Age	− 0.24 (−0.56, 0.14)	0.208		
Sex (0 = male, 1 = female)	0.14 (−0.24, 0.48)	0.470		
PBT sessions attended	− 0.16 (−0.51, 0.23)	0.397		
Treadmill experience	0.25 (−0.13, 0.57)	0.187		
Education	− 0.37 (−0.65, 0.00)	0.051		
Fall history	**− 0.43 (−0.69**,** − 0.07)**	**0.021**	**−0.38 (−0.71**,** −0.02)**	**0.038**
Fried frailty phenotype (score)	− 0.21 (−0.54, 0.18)	0.275		
Mini-Mental State Examination	0.13 (−0.25, 0.47)	0.512		
Trail Making Test B-A	− 0.04 (−0.40, 0.33)	0.836		
Short Falls Efficacy Scale-International	**− 0.48 (−0.72**,** − 0.12)**	**0.009**	−0.28 (−0.61, 0.07)	0.116
Timed Up and Go	− 0.18 (−0.52, 0.20)	0.339		
Short Physical Performance Battery	0.16 (−0.23, 0.51)	0.398		
4-m gait speed test	0.11 (−0.28, 0.47)	0.579		
2-min walk test	0.25 (−0.13, 0.57)	0.190		
Brief Balance Evaluation Systems Test	0.01 (−0.38, 0.38)	0.980		
Four Square Step Test	− 0.25 (−0.57, 0.14)	0.188		
Stepping Threshold Test (ACE)	0.14 (−0.25, 0.49)	0.480		
Stepping Threshold Test (DSE)	0.27 (−0.12, 0.58)	0.170		
Dynamic Stepping Threshold Test	0.23 (−0.16, 0.55)	0.244		

^1^The multivariable regression model (enter method) only included participant characteristics showing significant correlations in (*p* <.05) in the bivariate analysis. The final model was significant (*F*(2,26) = 4.49, *p* =.021), explaining 20% of the variance (adjusted *R²*=0.20). Correlation coefficients (*r*) reported for point-biseral (sex, treadmill experience, fall history), Spearman (PBT sessions attended, Fried frailty phenotype, Short Falls Efficacy Scale-International, Timed Up and Go, Short Physical Performance Battery, 4-m gait speed test, Four Square Step Test), or Pearson correlations (age, education, Mini-Mental State Examination, Trail Making Test B-A, 2-min walk test, Brief Balance Evaluation Systems Test, [Dynamic] Stepping Threshold Test). ACE = all-step count evaluation, *β*_*std*_ = standardized regression coefficient, CI = confidence interval, DSE = direction-sensitive evaluation

### Focus groups

Focus group participants did not differ significantly from non-participants in descriptive measures, number of PBT sessions attended, or TFA questionnaire scores (*p* =0.170-0.998), except that they showed a higher gait speed (*p* =0.045). Brief information on the characteristics of focus group participants and trainers is provided in Table 5. Qualitative findings from participant and trainer focus groups are presented along the interview guide structure, with additional supporting quotes in Supplementary Table 4.

**Table 5:** Information on focus group attendees

Attendees (ID)	Sex	Age (years)	Fall history	Concerns about falling	Arm
P03	F	79	N	Moderate	2PBT
P04	F	71	Y	Moderate	2PBT
P05	M	83	Y	Moderate	6PBT
P06	M	85	N	Moderate	2PBT
P08	F	71	N	High	6PBT
P12	F	83	N	Moderate	6PBT
P24	M	83	N	Low	6PBT
P28	F	80	N	Low	6PBT
P31	F	70	Y	Moderate	6PBT
P32	F	77	N	Low	2PBT
P33	F	81	N	Low	2PBT
P35	M	79	N	Moderate	6PBT
T01	F	22	N	-	T
T02	F	23	N	-	T
T03	F	21	N	-	T

F = female, ID = identification number, M = male, N = no, P = participant, T = trainer, Y, yes, 2PBT = two-session perturbation-based balance training and four-session conventional treadmill training, 6PBT = six-session perturbation-based balance training. Concerns about falling were categorized as low (7–8 pt.), moderate (9–13 pt.), high (14–28 pt.) according to the Short Falls Efficacy Scale-International

### Affective attitude

All participants perceived PBT as novel and engaging, with some describing it as comfortable and even enjoyable.

*“For me*,* it was totally exciting*,* like a little adventure. The situations where I wasn’t under control were*,* let’s say*,* the icing on the cake and made it really fun.” (P24)*.

In contrast, other participants were relieved when PBT ended, reporting tension during sessions that caused discomfort and occasionally even triggered memories of past falls.

*“It wasn’t fun. It really strained my nerves. I was constantly tense*,* waiting for the next perturbation.” (P04)*.

Trainers, delivering PBT for the first time, reported a positive atmosphere, good feasibility, and impressive improvements in participants’ reactive balance.

*“I found it to be a very individualized training […]*,* and I personally was positively surprised by what they learned and how they performed after the six weeks.” (T01)*.

### Burden

Some participants found PBT acceptably challenging or strenuous, while others experienced it as very demanding both physically and mentally.

*“Despite everything*,* I have to say it was challenging. […] I couldn’t handle it lightly. […] The half-hour pushed me to my limits. I found it exhausting; it was a completely new experience for me.” (P31)*.

They reported feeling physically exerted and sweaty, highly concentrated and focused, and stressed particularly by the uncertainty of upcoming perturbations.

*“Yes*,* a little [burdensome]*,* because it’s unpredictable. You’re occupied with what’s coming next*,* especially when all four directions are involved*,* and you don’t know which direction will come. But that’s just how it is—you don’t know. So*,* you end up focusing on when it’s going to happen.” (P32)*.

Trainers initially had some safety concerns, but these subsided quickly. They did not find delivering PBT burdensome or challenging, though they reported some initial caution.

*“If something had happened*,* we would have been somewhat at fault*,* and that was one of my initial concerns. But it became clear relatively quickly that the administration was quite simple and that nothing was really likely to go wrong.” (T02)*.

### Ethicality

Many participants generally agreed that PBT matched their expectations of fall prevention. A few found it difficult to articulate such expectations but valued the opportunity to contribute to science.

*“[…] based on my reading experience*,* a fall prevention program should really include at least balance and strength training*,* actually those two components. I already do strength training all the time in REGE e.V.*,* so it [PBT] makes sense to me.” (P06)*.

Trainers shared this general view but also recognized the potential of PBT as an innovative reactive training approach to implement.

*“I think if I would have been thinking about fall prevention in general*,* I probably wouldn’t think of something like this. However*,* I find it to be a pretty good approach to really challenge reactive balance.” (T01)*.

### Intervention coherence

Most participants were able to describe the aim of the PBT, primarily summarizing what it consists of.

*“What you wanted to achieve: simply handling unforeseen situations*,* getting them under control*,* as one would say.“ (P08)*.

Others described in more detail the specific mechanisms PBT aimed to improve.

*“So*,* it was meant to challenge stumbling*,* so that you support yourself*,* catch yourself*,* absorb the impact - however one might express it.” (P04)*.

A few participants raised concerns about the ecological validity of PBT, noting that it lacked real-life distractions that can make quick reactions harder. Both participants and trainers also remarked on the absence of obstacles to trip over.

*“What was really missing was something to actually trip over. I would definitely support including that.” (T02)*.

Trainers also clearly understood the aim of PBT and were able to describe it as task-specific, effectively targeting the intended skills, and well-suited to challenge reactive balance.

*“I would say the ability to react to unexpected perturbations*,* in these four directions*,* to respond better and ultimately avoid a fall.” (T02)*.

### Perceived effectiveness

Many participants reported daily-life benefits from PBT, most often describing a more stable walking and improved balance reactions, while only a few did not.

*“I definitely noticed improvements*,* and even my wife said I walk better.”* (P06).

*“It helped me. I’ve been in near-fall situations several times since then and I reacted well – nothing happened.” (P24)*.

Trainers were positively surprised by the participants’ progress. They noticed not only improved recovery from (more intensive) perturbations and greater ease of walking, but also reduced anxiety and even casual conversations during PBT.

*“Overall*,* I think they gained a lot from it - especially those who were really engaged with it. Some even approached us on their own and said they really noticed the effects in their daily lives.” (T01)*.

### Self-efficacy

All participants felt that they had performed well, expressing satisfaction with how they managed PBT.

*“Well*,* I think I did quite well. I wouldn’t say it’s my favorite thing to do*,* but I managed*,* and I think it was pretty good.” (P04)*.

Even a very anxious participant was positive about her performance.

*“Yes*,* despite my excessive fear*,* I felt that*,* in my opinion*,* I still did quite well.” (P31)*.

Trainers felt confident in delivering PBT safely and effectively after becoming familiar with the setting.

*“With routine it became a good process - and then you did one training after another. […] By the end*,* once you got used to it*,* it felt good.“ (T01)*.

### Opportunity costs

Participants did not report having to forgo other activities, as PBT was integrated in their regular REGE e.V. training sessions. However, some would have preferred longer overall training time rather than replacing other exercises with PBT.

*“If that was integrated [in the REGE e.V. session]*,* the duration would need to be extended. Maybe 20 more minutes*,* or even two hours in total instead of just one and a half.” (P04)*.

### Training setting

The perturbation treadmill was perceived as impressive but intimidating due to its elevated platform and missing handrails. Some participants described treadmill walking as monotonous, with one suggesting adding video content to PBT sessions. While 2PBT participants wished for more sessions, those in the 6PBT group were satisfied. All participants agreed that 30 min per session was appropriate and sufficient.

*“So*,* it’s not really my thing [the PBT treadmill]*,* a bit boring there with the device.” (P04)*.

*“I actually found it quite reasonable. You could have done it a little longer*,* but if you are supposed to react properly*,* then you can’t extend it for too long.” (P06)*.

The majority felt very comfortable with 1:1 supervision and preferred individual PBT. However, some missed social interaction with peers and suggested that PBT alongside others might be helpful, especially for those experiencing anxiety.

*“I would have liked to see how others did it. […] I think seeing someone else do it without fear could be helpful for someone who is anxious.” (P03)*.

Trainers also valued 1:1 supervision but acknowledged that this preference might be highly individual. They noted that group training could motivate some participants but also prompt overestimating of abilities in others.

*“Since it was a bit tricky for participants at the beginning*,* it was good that PBT was done individually.” (T01)*.

*“I could imagine it being motivating if several people could train at the same time. I’m not sure whether that would be too distracting or make people feel insecure because someone else is there. […] I don’t think you can generalize.” (T02)*.

#### Tailoring

Participants found PBT well adapted to their abilities, without feeling under- or overchallenged. The rating of anxiety and difficulty on the Likert scales was perceived as difficult and negatively associated with medical visits.

*“Well*,* despite everything*,* it was definitely an effort. I didn’t feel underchallenged.” (P04)*.

Trainers confirmed that adjusting the training intensity based on participants’ ratings was not easy. In their opinion, participants should have been challenged slightly more to really train at the limits of stability.

*“I think*,* in some cases*,* participants’ self-ratings of anxiety and difficulty levels were not entirely reliable*,* as they themselves were unsure about how to express their feelings.” (T02)*.

### Anxiety and safety

Anxiety was experienced differently. Some participants described considerable fear (of falling) that diminished with practice, while for others it increased through anticipation of upcoming perturbations. However, most reported not anxiety but rather tension.

*“You were safe because of being suspended*,* so there was no need to be afraid*,* or at least I wasn’t afraid - that might be the best way to say it.” (P12)*.

*“In the beginning*,* it was exhausting due to the uncertainty that something could happen. That subsided over time. In the end*,* I was completely stress-free.” (P05)*.

All participants felt safe, including those who were anxious. Their sense of safety was mainly attributed to the safety harness and the trainers’ attentive supervision.

*“There was actually also a sense of safety - if I stumble*,* I know I won’t fall. So*,* nothing can happen to me.” (P24)*.

The harness protection also supported the trainers’ perception of PBT as safe. However, they noted that the treadmill belt was very narrow, and participants’ anxiety acted as a limiting factor for conducting PBT.

*“I think that the higher the level of fear*,* the less likely someone would participate. Therefore*,* sufficient preparatory work is needed to convince them that nothing can happen*,* that everything is secured*,* and that the training will be stopped if their fear becomes too overwhelming*,* […].” (T01)*.

## Discussion

This study is the first to explore the acceptability of PBT from both participant and trainer perspectives using a mixed-methods approach. Overall, findings indicate that treadmill PBT was well accepted by older adults at risk of falling and by trainers. The integration of quantitative and qualitative data provides robust support for its use in fall prevention, while also highlighting specific challenges, particularly related to fall history and anxiety, that may limit acceptability.

### Overall acceptability and fall history

Findings of the TFA questionnaire demonstrated high overall acceptability among participants, consistent with previous qualitative surveys in less physically impaired older adults [9]. Although fall history emerged as negative predictor, participants with prior falls still rated treadmill PBT as moderately to highly acceptable. This aligns with previous qualitative interviews showing also good acceptability in older adults with a recent fall history [8]. Lower acceptability in this subgroup may reflect greater concerns about falling and activity avoidance after prior falls [38, 39], reinforced by the near-fall exposure inherent to PBT [40]. Indeed, some participants described perturbations as anxiety-inducing or reminiscent of past falls. Fall history, however, did not uniformly reduce acceptability, suggesting individual variability that may relate to the cause of previous falls. Slip- or trip-related falls may more easily trigger memories than medical causes (e.g., dizziness, medical events), an aspect warranting further investigation. Overall, findings suggest that treadmill PBT may be most acceptable as a primary fall-prevention strategy, ideally implemented before the first fall to minimize fall-related anxiety.

### Anxiety, safety, and affective attitude

Anxiety is a well-documented side effect and potential barrier of PBT [8, 10, 15, 41, 42], more frequent than in other interventions [43], and a practical challenge for implementation [6, 44].

Such anxiety may also be associated with heightened concerns about falling, reduced confidence, and avoidance behaviors, which may reduce engagement and adherence within PBT and potentially extend to activity avoidance beyond the training context. In our study, participants often reported tension rather than overt anxiety; while some adapted over time, others experienced persistent or increasing anxiety. As uncertainty is known to heighten anxiety during PBT [10], the high unpredictability combined with relatively intense perturbations in our protocol may have reinforced these responses. A more gradual progression in unpredictability (e.g., verbal or visual cues on perturbation timing and/or direction) and intensity may improve acceptability in future PBT protocols by allowing participants to acclimate [6] and mitigating these adverse psychological impacts.

Although monitoring anxiety with numerical scales has been proposed as a facilitator for alleviating it [10], our participants found this approach challenging, likely due to difficulties in translating subjective experiences into scores, as also reported previously [8]. Only one dropout was attributed to anxiety, and trainers noted that it generally decreased over time, suggesting it was successfully managed. Tailoring, individualized progression, and 1:1 supervision, core elements of our PBT protocol, may have supported this process, as these have been recognized as key factors for reducing anxiety in PBT [8, 9, 15]. Additional facilitators included familiarization, trainer support, and the safety harness which likely helped build rapport and trust between trainers and participants, aspects considered crucial for alleviating anxiety [42, 44]. Nevertheless, trainers regarded participants’ anxiety a limiting factor not only for acceptability but also for feasibility and effectiveness, confirming its negative impact on reactive balance through delayed and more rigid responses [45, 46].

Affective attitudes toward PBT were largely positive, with many participants describing it as novel, engaging, and even fun, aligning with previous treadmill PBT studies [8, 9, 15]. However, a few reported discomfort or relief when PBT ended, an observation not noted in prior studies and possibly related to the lower physical capacity of our sample.

Importantly, despite some anxiety and negative affective attitudes, participants and trainers consistently reported a strong sense of safety, largely attributed to the harness and close supervision. This aligns with previous findings showing that these factors provide a safe training environment for treadmill PBT [8], even for anxious individuals [9, 15]. “Testing” the harness through full-body suspension may also have reinforced safety perceptions, as reported for other challenging balance exercises in older adults [47]. In contrast, the narrow treadmill belt was mentioned by trainers as a safety concern, which may reflect a limitation of the specific perturbation treadmill used and/or the lower physical capacity level our sample (e.g., wider step width) compared with studies where this issues were not raised [15].

Implementation of PBT in real-world settings should account for anxiety as a key potential barrier, emphasizing the importance of fostering a strong sense of safety for participants. Key considerations include adequate familiarization, as well as tailoring and progression of perturbations (i.e. unpredictability and intensity), and close supervision within a safe training environment. This requires appropriately trained personnel and suitable safety equipment.

### Perceived demands, tailoring, and self-efficacy

Knowledge about dose-response relationship of PBT in older adults, particularly those at risk of falling, remains inconclusive [6]. As with other exercise, benefits occur only when training intensity exceeds individual thresholds. In this study, intensity was individually adjusted to participants’ self-perceived difficulty and anxiety, aiming for moderate levels to provide an effective yet manageable stimulus. Moderate burden ratings suggest that tailoring was successful and that PBT was not perceived as overly demanding when adapted accordingly, consistent with previous treadmill PBT studies [8, 9]. Focus group statements supported this, noting that while some participants found PBT physically and mentally demanding, mainly due to the unpredictability of perturbations, others considered the challenge acceptable. Trainers also reported moderate burden and emphasized that tailoring based solely on participants’ perceptions was challenging, as these often misaligned with individual thresholds. Future protocols may therefore benefit from relying more strongly on trainer expertise while still incorporating participant feedback to guide tailoring, individualization, and progression.

Despite its challenges, both participants and trainers perceived PBT as manageable. This likely reflects the role of tailoring, individualized progression, and 1:1 supervision, as self-efficacy can be strengthened through encouragement, guidance, and trust in trainers during challenging balance exercises [44, 47]. Trainers also valued this individualized environment and reported confidence in delivering treadmill PBT independently, consistent with findings from other healthcare professionals [15].

### Intervention coherence and perceived effectiveness

Both participants and trainers demonstrated a clear understanding of the aims and rationale of PBT. Some even described the specific mechanisms targeted, underscoring its transparency and task relevance. This aligns with previous studies showing that treadmill PBT has an easily communicated rationale [8, 15]. Suggestions to incorporate real-life elements such as distractions or obstacles may further improve ecological validity of PBT. Overall, strong intervention coherence appears as important strength of PBT, as its rationale is both understandable and communicable, potentially supporting information and recruitment processes for this fall-prevention training.

Perceived effectiveness was also rated very high by both participants and trainers, indicating that PBT was not only well understood but also regarded as beneficial for improving fall-resisting skills in daily life. This is encouraging, as improvements in reactive balance [43] and self-perceived balance ability have both been associated with lower fall risk [48] Similar findings have been reported in previous treadmill PBT studies, where older adults described improved walking ability and balance confidence/awareness [8, 15]. These findings suggest that the effectiveness of PBT was demonstrated not only objectively in the FEATURE trial [22] but also subjectively, as directly perceived by participants.

## Ethicality, opportunity costs and training setting

PBT aligned well with the values and expectations of participants and trainers, who regarded it as a meaningful contribution to fall prevention. For participants, this likely reflected their strong fall awareness, fostered by regular engagement in fall prevention at REGE e.V., a factor previously identified facilitating ethicality perceptions in PBT [15]. Trainers emphasized PBT’s innovative, task-specific nature as a valuable complement to traditional exercises, reinforcing its practical relevance.

Opportunity costs appeared low, as participants had no concerns about replacing parts of their usual training with PBT. For the long term, however, several preferred extending overall training duration rather than replacing existing exercises, suggesting that PBT may be best positioned as an addition within multi-component fall-prevention programs. This highlights the importance of exercise variety and sufficient training volume for sustaining motivation and long-term engagement.

The training setting also shaped acceptability. Although participants favored individual delivery, consistent with prior findings [8, 15], they also valued the social interaction of group-based sessions. As social aspects are known facilitators of fall-prevention programs [8, 49], combining individualized PBT with group-based exercise could be a promising future approach.

The perturbation treadmill, previously described as facilitator of PBT [8], was viewed more ambivalently in our study, mainly due to its elevated platform and lack of handrails, barriers to also noted previously [15, 50]. Treadmill walking itself may pose additional challenges for populations at risk of falling [6, 22], suggesting that alternative PBT paradigms such as walkways, which appear less anxiety-inducing [50], could offer potential advantages for these groups.

Overall participant acceptability showed no association with the number of PBT sessions at-tended in quantitative analyses, consistent with our previous findings [22]. In contrast, qualitative analyses suggest that receiving more than two PBT sessions may positively contribute to acceptability. While 30-min PBT sessions were generally perceived acceptable, some participants found treadmill walking monotonous and boring, as also reported elsewhere [51]. Suggested improvements included adding video content (e.g., nature walks, city tours) to sustain engagement and further enhance ecological validity by providing realistic distractions. Studies combining treadmill PBT with virtual reality [8] or playful dual-task elements [9] reported high acceptability, with older adults describing PBT as positive, surprising, and fun, without monotony.

### Strengths and limitations

The main strength of this study lies in its convergent mixed-methods design [20], combining quantitative and qualitative data and integrating both at the interpretation stage to provide a more comprehensive understanding of participants’ and trainers’ PBT acceptability. However, several limitations should be noted. First, the study was conducted within a senior fitness club, limiting generalizability to other contexts (e.g., clinical settings) or older adults not regularly engaged in exercise. Second, the qualitative feedback from the three trainers reflects a limited range of perspectives and may not fully represent the diversity of experiences that could emerge in larger or more heterogeneous trainer samples, thereby precluding definitive conclusions. Third, sport science students acted as trainers, which may not reflect the perspectives of more experienced trainers delivering PBT in clinical settings. Fourth, a self-developed TFA questionnaire was used, as the validated generic version was not yet available [52]. Fifth, no acceptability data was available from participants who withdrew from or did not begin PBT. Finally, findings are specific to treadmill PBT and may not apply to other modalities (e.g., walkways).

## Conclusion

Treadmill PBT was well accepted by both older adults at risk of falling and trainers, with lower acceptability among participants with a history of falls. High perceived safety and effectiveness in improving fall-resisting skills, adequate tailoring and supervision, and strong coherence contributed positively to acceptability. Anxiety, fall-related memories, the demanding nature of PBT, and training-specific factors such as treadmill monotony, limited social interaction during individual sessions, and device-related characteristics (missing handrails, narrow belt) contributed negatively to acceptability. Overall, implementing PBT in individuals with no fall history may help mitigate anxiety related to prior fall experiences, thereby maximizing both acceptability and PBT-related benefits.

## Supplementary Information

Below is the link to the electronic supplementary material.

Supplementary Material 1

## Data Availability

The data sets used and/or analyzed during the current study are available from the corresponding author upon reasonable request.
