# Supplementary material for: Acceptability of treadmill perturbation-based balance training in older adults at risk of falling: A mixed-methods evaluation of participant and trainer perspectives
Source: Aging Clin Exp Res. 2026 Jan 16;38(1):69. doi: 10.1007/s40520-025-03320-0 (PMC12886240; doi:10.1007/s40520-025-03320-0)
Supplement: Supplementary file 1 — Supplementary Material 1 [file 40520_2025_3320_MOESM1_ESM.docx]

**Supplementary Table 1.** Good reporting of a mixed-methods study (GRAMMS) checklist

| **No.** | **Reporting item** | **Section, page information** |
| --- | --- | --- |
| 1 | Describe the justification for using a mixed methods approach to the research question | Introduction  Discussion |
| 2 | Describe the design in terms of the purpose, priority, and sequence of methods | Methods – Study design  Methods – Acceptability measures  Discussion |
| 3 | Describe each method in terms of sampling, data collection and analysis | Methods – Acceptability measures  Methods – Statistical and analytic procedures |
| 4 | Describe where integration has occurred, how it has occurred and who has participated in it | Methods  Discussion |
| 5 | Describe any limitation of one method associated with the present of the other method | Discussion |
| 6 | Describe any insights gained from mixing or integrating methods | Discussion |

**Reference**

O'Cathain A, Murphy E, Nicholl J. The quality of mixed methods studies in health services research. J Health Serv Res Policy. 2008;13: 92-98.

**Supplementary Table 2.** Consolidated criteria for reporting qualitative research (COREQ) checklist

| **No.** | **Item** | **Guide question/description** |
| --- | --- | --- |
| **Domain 1: Research team and reflexivity** | | |
| *Personal characteristics* | | |
| 1 | Interviewer/facilitator | First author **(Methods, Focus groups)** |
| 2 | Credentials | M.Sc. (first and second author) |
| 3 | Occupation | PhD student (first author) and research associate (second author) |
| 4 | Gender | Female (first and second author) |
| 5 | Experience and training | Coursework and mentoring by experienced qualitative researchers (first and second author) |
| *Relationship with participants* | | |
| 6 | Relationship established | Relationship was established within the role of FEATURE study coordination via phone and in-house screening for participants and as (thesis) supervisor for trainers.  **(Methods, Focus groups)** |
| 7 | Participant knowledge of the interviewer | Participants and trainers were informed of the first author’s purpose of the study. |
| 8 | Interviewer characteristics | The reason and interest in the topic were shared with the participants and trainers. |
| **Domain 2: Study design** | | |
| *Theoretical framework* | | |
| 9 | Methodological orientation and theory | Theoretical framework of acceptability (TFA)  **(Methods, Focus groups)** |
| *Participant selection* | | |
| 10 | Sampling | Convenience sampling **(Methods, Focus groups)** |
| 11 | Method of approach | All participants were approached when completing the PBT intervention. Interested participants contacted the researchers via phone. Trainers were approached at the end of the intervention. **(Methods, Focus groups)** |
| 12 | Sample size | 15 (12 participants, 3 trainers) **(Results, Participant and trainer characteristics, and Focus groups)** |
| 13 | Non-participation | Not applicable |
| *Setting* | | |
| 14 | Setting of data collection | Clinic **(Methods, Focus groups)** |
| 15 | Presence of non-participants | No one was present besides the researchers. |
| 16 | Description of sample | 12 participants, predominantly female (67%) with fall history in *n*=3. Trainers were female sports science students. **(Results, Participant and trainer characteristics, and Focus groups)** |
| *Data collection* | | |
| 17 | Interview guide | Focus group themes and questions guided by the TFA were provided **(Table 1)**, according to Gerards et al. (2022) why it was not piloted. |
| 18 | Repeat interviews | Not applicable |
| 19 | Audio/visual recording | Focus groups were audio recorded. **(Methods, Focus groups)** |
| 20 | Field notes | Field notes were taken during the focus groups by the second author. **(Methods, Focus groups)** |
| 21 | Duration | Approximately 60 minutes per focus group. **(Methods, Focus groups)** |
| 22 | Data saturation | Not formally assessed; pilot study with predefined sample size and framework-based data collection. |
| 23 | Transcripts returned | Transcripts were not returned to participants/trainers for comment and/or correction. |
| **Domain 3: Analysis and findings** | | |
| *Data analysis* | | |
| 24 | Number of data coders | Coding was done by first author. **(Methods, Statistical and analytical procedures)** |
| 25 | Description of the coding tree | No coding tree was used. |
| 26 | Derivation of themes | Findings were derived deductively guided by the TFA domains and other pre-identified PBT-related topics. **(Methods, Statistical and analytical procedures)** |
| 27 | Software | Not applicable; manual analysis |
| 28 | Participant checking | Participant feedback on the findings was not used. |
| *Reporting* | | |
| 29 | Quotations presented | Findings are illustrated by representative quotes from participants and trainers. Each quotation was identified by participant/trainer number. **(Results, Focus groups)** |
| 30 | Data and findings consistent | There is consistency between the data presented and the findings. |
| 31 | Clarity of major themes | Major themes are clearly presented along the TFA domains and context-specific topics. **(Results, Focus groups)** |
| 32 | Clarity of minor themes | Not applicable |

**Reference**

Tong A, Sainsbury P, Craig J. Consolidated criteria for reporting qualitative research (COREQ): a 32-item checklist for interviews and focus groups. Int J Qual Health Care. 2007;19(6):349-357.

**Supplementary Table 3.** Acceptability questionnaire based on the Theoretical Framework of Acceptability (according to Sekhon et al.)

|  | **Statement** | **Strongly disagree** | **Disagree** | **Neither**  **agree nor disagree** | **Agree** | **Strongly**  **agree** |
| --- | --- | --- | --- | --- | --- | --- |
| 1 | The PBT met my expectations of an effective training to prevent a fall in everyday life. | ❑  (1) | ❑  (2) | ❑  (3) | ❑  (4) | ❑  (5) |
| 2 | In my opinion, participation in PBT required a large amount of effort. | ❑  (5) | ❑  (4) | ❑  (3) | ❑  (2) | ❑  (1) |
| 3 | In my opinion, the PBT was useful to improve my ability to prevent a fall in everyday life. | ❑  (1) | ❑  (2) | ❑  (3) | ❑  (4) | ❑  (5) |
| 4 | I felt comfortable during the PBT. | ❑  (1) | ❑  (2) | ❑  (3) | ❑  (4) | ❑  (5) |
| 5 | In my opinion, the PBT can be effective to prevent a future fall in everyday life. | ❑  (1) | ❑  (2) | ❑  (3) | ❑  (4) | ❑  (5) |
| 6 | PBT was mostly an overload for me. | ❑  (5) | ❑  (4) | ❑  (3) | ❑  (2) | ❑  (1) |
| 7 | I had no problem replacing a part of my normal training to attend the PBT. | ❑  (1) | ❑  (2) | ❑  (3) | ❑  (4) | ❑  (5) |
| PBT = perturbation-based balance training. Statements according to the Theoretical Framework of Acceptability domains: 1=ethicality, 2=burden, 3=intervention coherence, 4=affective attitude, 5=perceived effectiveness, 6=self-efficacy, 7=opportunity costs. | | | | | | |

**Note**: Trainer questionnaire was adjusted by adapting question 2 to “In my opinion, delivering PBT required a large amount of effort”, question 3 to “In my opinion, the PBT was useful to improve the ability to prevent a fall in everyday life”, question 6 to “Delivering PBT was mostly an overload for me”, and removing question 7.

**Reference**

Sekhon M, Cartwright M, Francis JJ. Development of a theory-informed questionnaire to assess the acceptability of healthcare interventions. BMC Health Serv Res. 2022;22(1):279.

**Supplementary Table 4.** Participant and trainer quotes

| **TFA domain** | **Participant** | **Trainer** |
| --- | --- | --- |
| Affective  attitude | - “For me, it was totally exciting, like a little adventure. The situations where I wasn’t under control were, let’s say, the icing on the cake and made it really fun.” (P35) - What I can say is that I liked it and that it was good for me. I noticed that my reactions and responsiveness were quite good, and I liked that.” (P24) - “I also thought it would be really nice if we would have the device in our group [REGE].” (P12) - “It wasn’t fun. It really strained my nerves. I was constantly tense, waiting for the next perturbation.” (P04) - “I must say, I don’t find the perturbations very pleasant.” (P32) | - "I found it to be a very individualized training [...], and I personally was positively surprised by what they learned and how they performed after the six weeks." (T01) |
| Burden | - "Despite everything, I have to say it was challenging. [...] I couldn’t handle it lightly. [...] The half-hour pushed me to my limits. I found it exhausting; it was a completely new experience for me." (P31) - “Yes, a little [burdensome], because it's unpredictable. You're occupied with what’s coming next, especially when all four directions are involved, and you don't know which direction will come. But that's just how it is—you don’t know. So, you end up focusing on when it’s going to happen.” (P32) - “In terms of attention, it definitely challenged me.” (P06) - “I noticed that I was really sweating by the end, and that’s when you realize how demanding it was.” (P12) | - “If something had happened, we would have been somewhat at fault, and that was one of my initial concerns. But it became clear relatively quickly that the administration was quite simple and that nothing was really likely to go wrong.” (T02) - “But I think that’s always the case at the beginning—until you get a bit used to the equipment. I did find the setup challenging at first.” (T01) |
| Ethicality | - “[…] based on my reading experience, a fall prevention program should really include at least balance and strength training, actually those two components. I already do strength training all the time in REGE e.V., so it [PBT] makes sense to me.” (P06) | - “I think if I would have been thinking about fall prevention in general, I probably wouldn’t think of something like this. However, I find it to be a pretty good approach to really challenge reactive balance.” (T01) |
| Intervention  coherence | - “What you wanted to achieve: simply handling unforeseen situations, getting them under control, as one would say.“ (P08) - "So, it was meant to challenge stumbling, so that you support yourself, catch yourself, absorb the impact - however one might express it.” (P04) | - "What was really missing was something to actually trip over. I would definitely support including that.” (T02) - "I would say the ability to react to unexpected perturbations, in these four directions, to respond better and ultimately avoid a fall." (T02) - “I think it’s a very good way to really challenge reactive balance, because when I see an obstacle, I can prepare myself better. Of course I still have to react to the balance disturbance, but not knowing in which direction something will come is very specific and brings out exactly what the training aims to target.” (T03) |
| Perceived  effectiveness | - "I definitely noticed improvements, and even my wife said I walk better." (P06) - "It helped me. I’ve been in near-fall situations several times since then and I reacted well – nothing happened." (P24) - “After the perturbations, not immediately on the same day but maybe one or two days later, I had the feeling that I had more strength in my legs. I felt more secure on my feet.” (P04) - “I notice it at home. When I used to stumble, I let out a loud scream out of shock and react quite awkwardly, but now when something like that happens, I’m no longer nearly as afraid.” (P08) | - “Overall, I think they gained a lot from it - especially those who were really engaged with it. Some even approached us on their own and said they really noticed the effects in their daily lives.” (T01) - “Just by having that progression [of the perturbation intensity], you could already notice the improvement.” (T02) - “What stood out was that toward the end, some of them started talking during the training and became much more relaxed in getting back into normal gait.” (T01) |
| Self-efficacy | - "Well, I think I did quite well. I wouldn’t say it’s my favorite thing to do, but I managed, and I think it was pretty good." (P04) - “Yes, despite my excessive fear, I felt that, in my opinion, I still did quite well." (P31) | - “With routine it became a good process - and then you did one training after another. [...] By the end, once you got used to it, it felt good.“ (T01) |
| Opportunity costs | - "If that was integrated [in the REGE e.V. session], the duration would need to be extended. Maybe 20 more minutes, or even two hours in total instead of just one and a half." (P04) - “It was just a matter of planning. For me, it meant blocking it in my calendar and allowing enough time so that it would work. So I think, yes, it was mainly about planning.” (P12) - “I would do it once a month. I wouldn’t like to do it once a week.” (P35) | - |
| **Context-specific factors** | | |
| Training  setting | - “So, it’s not really my thing [the PBT treadmill], a bit boring there with the device.” (P04) - "I actually found it quite reasonable. You could have done it a little longer, but if you are supposed to react properly, then you can't extend it for too long." (P06) - "I would have liked to see how others did it. [...] I think seeing someone else do it without fear could be helpful for someone who is anxious." (P03) - “You also get distracted by the other person, watching how they do it, whether they have the same difficulties or do it better, and are much older.” (P04) - “I could also imagine doing it in a group, but that would mean someone else would need to be there to supervise it. If you feel comfortable in a group, it’s nice to do it together and have that exchange.” (P08) - “I think it would be more distracting if other people were next to me. I concentrate so much on my legs and on the perturbation that I would not want to be distracted from the sides. For me, doing it individually was actually quite good.” (P32) | - “Since it was a bit tricky for participants at the beginning, it was good that PBT was done individually.” (T01) - “I could imagine it being motivating if several people could train at the same time. I’m not sure whether that would be too distracting or make people feel insecure because someone else is there. […] I don’t think you can generalize.” (T02) |
| Tailoring | - “Well, despite everything, it was definitely an effort. I didn’t feel underchallenged.” (P04) - “By the end, I really felt challenged.” (P35) - “Sometimes I found it difficult to rate it [difficulty and anxiety] on a scale from one to five.” (P12) | - "I think, in some cases, participants' self-ratings of anxiety and difficulty levels were not entirely reliable, as they themselves were unsure about how to express their feelings.” (T02) - “Some of them said they didn’t find it difficult at all, but on the other hand you could clearly see that there was some difficulty, and also the fear was completely manageable, but you could see that they were still a bit tense.” (T03) |
| Anxiety and safety | - “You were safe because of being suspended, so there was no need to be afraid, or at least I wasn’t afraid - that might be the best way to say it." (P12) - "In the beginning, it was exhausting due to the uncertainty that something could happen. That subsided over time. In the end, I was completely stress-free." (P05) - “I wasn’t afraid, but I felt challenged.” (P06) - “I became increasingly afraid with each new session. The first two or three times were still okay. I had to concentrate intensely, and after the fourth and fifth session, I think I didn’t complete the training anymore […] because my fear kept increasing.” (P31) - "There was actually also a sense of safety - if I stumble, I know I won’t fall. So, nothing can happen to me." (P24) - “I never thought about a fall, I knew I would be caught, and then I’d just be hanging in the air.” (P12) | - "I think that the higher the level of fear, the less likely someone would participate. Therefore, sufficient preparatory work is needed to convince them that nothing can happen, that everything is secured, and that the training will be stopped if their fear becomes too overwhelming, […]." (T01) - “They [participants] often said they wouldn’t describe it as fear, but rather as a kind of tension.” (T02) - “I would generally say it [anxiety] is definitely something inhibiting.” (T01) - “I felt that for some [participants] it helped to repeat it again and again, but others remained anxious, I would say.” (T03) - “I actually found it very safe, and because of the harnesses they [participants] were secured from top to bottom, so I didn’t feel that anything serious could really happen.” (T01) |

**Supplementary Material 5.** Descriptive measures

Sociodemographic and clinical characteristics including age, sex, years of education, treadmill experience (yes vs. no), and fall history in the last 12 months (yes vs. no) were assessed based on participants’ self-report.

Fried frailty phenotype was assessed using five criteria (Fried et al., 2001): (1) self-reported unintentional weight loss (>4.5 kg in the past year); (2) self-reported exhaustion (2-items from the Center for Epidemiological Survey-Depression Scale); (3) low physical activity (short version of the Minnesota Leisure Time Physical Activity Questionnaire: female < 270 kcal/week, male < 383 kcal/week); (4) slow gait speed (gender- and height-adjusted cut-offs), and (5) weakness (gender- and body-mass-index-adjusted low maximum handgrip strength as measured with a JAMAR digital hand dynamometer). A Fried frailty phenotype score was calculated based on the number of criteria met (range: 0-5), with participants classified as robust (0 criteria), pre-frail (1-2 criteria), or frail (≥3 criteria). It demonstrates concurrent and predictive validity for the adverse outcomes that geriatricians identify frail older adults as being at risk for falls, hospitalizations, disability, and death (Fried et al., 2001).

Cognitive functioning was evaluated using the Mini-Mental State Examination (MMSE; Folstein et al., 1975) and the Trail-Making Test (TMT) A & B (Reitan et al., 1992).

The MMSE is one of the most established screening tools for assessing global cognitive status in older adults, evaluating orientation, registration, attention, recall, language, and visuospatial abilities. MMSE scores range from 0 to 30 points, with higher scores indicating better cognitive functioning; scores of <24 points are indicative for cognitive impairment. The validity and reliability of the MMSE have been confirmed in numerous previous studies (Feeney et al., 2016; Folstein et al., 1975; Pangman et al., 2000; Tombaugh & McIntyre, 1992).

The TMT is one of the most established neuropsychological test for executive functioning. In part A, participants are required to connect numbers in ascending order as quickly as possible (processing speed, visual scanning), while in part B, participants alternate between numbers and letters in ascending order (cognitive flexibility, task shifting) (Reitan et al., 1992). Completion time in seconds is recorded for each part, with longer times indicating poorer executive functioning. The difference score (TMT B-A) is regarded as a refined measure of executive functioning, less influenced by visuo-perceptual and working memory demands (Sánchez-Cubillo et al., 2009). Normative values for TMT B-A values for German older populations are available, stratified by age and education (Specka et al., 2022). The validity and reliability of the TMT have been documented across diverse clinical, adult, and older populations (for a review, see Strauss et al., 2006).

Concern about falling was assessed using the Short Falls Efficacy Scale-International (Short FES-I) (Kempen et al., 2008), in which participants rate their level of concerns about falling during various activities of daily living. Short FES-I scores range from 7 to 28 points, with higher scores indicating greater concern about falling; scores are categorized as low (7-8 points), moderate (9-13 points), or high (14-28 points) concerns (Delbeare et al., 2010). The validity and reliability have been well-documented in previous studies (Delbeare et al., 2010; Hauer et al., 2010; Kempen et al., 2008).

Physical capacity was measured using the Timed Up and Go (TUG) (Podsiadlo & Richardson, 1991) and the Short Physical Performance Battery (SPPB) (Guralnik et al., 1994).

The TUG measures the time required for an individual to rise from a chair, walk 3 meters at a usual pace, turn around a cone, walk 3 meters back, and sit down on the chair. The TUG is recommended for assessing fall risk in older adults in the World Falls Guidelines (Montero-Odasso et al., 2022) and has well-established psychometric properties in this population (Lin et al., 2004; Podsiadlo & Richardson, 1991). TUG times of >12 seconds are indicative of an increased risk of falling (Lee et al., 2013).

The SPPB includes three tests of lower extremity function: a hierarchical standing balance test (Romberg, semi-tandem, and tandem stance), a usual gait speed test over 4 meters, and a 5-chair rise test (Guralnik et al., 1994). Each test is scored on a scale from 0 to 4 points, yielding a total score ranging from 0 to 12 points, with higher scores indicating greater physical capacity. SPPB scores of ≤9 points indicate an increased risk of future mobility disability (Vasunilashorn et al., 2009). The SPPB has been recommended by the World Health Organization (WHO) for assessing physical capacity (WHO, 2025), and its psychometric properties are well established among older adults (Freiberger et al., 2012; Kameniar et al., 2024).

Gait capacity was assessed using the 4-m usual gait speed test from the SPPB and the 2-min walk test (2MWT; Brooks et al., 2007). The 2MWT requires participants to walk as far as possible for 2 minutes along a flat 20-m corridor, turning back and forth at each end. The total distance walked is recorded, with greater walking distance indicating better gait capacity. Normative values for community-dwelling older adults have been reported to range from 134 to 155 m for women and from 144 to 184 m for men (Bohannon et al., 2015). The 2MWT has demonstrated good validity and reliability in older adults and across different patient populations (Bohannon et al., 2015; Brooks et al., 2006; Connelly et al., 2007; Pin, 2014).

Global balance was assessed using the Brief Balance Evaluation Systems Test (Brief-BESTest; Marques et al., 2016). The Brief-BESTest evaluates multiple components of balance across six domains: biomechanical constraints, stability limits, anticipatory postural adjustments, reactive postural responses, sensory orientation, and stability in gait. Each domain is represented by one item (two items with right and left components), scored on scale ranging from 0 to 3 points, yielding a total score ranging from 0 to 24 points, with higher scores indicating better global balance. Brief-BESTest scores of ≤12.5 points are indicative of an increased risk of falling (Marques et al., 2016). The validity and reliability of the Brief-BESTest for assessing balance in older adults and clinical populations have been demonstrated previously (Marques et al., 2016).

Dynamic balance was assessed using the Four Square Step Test (FSST; Dite & Temple, 2002). Participants are instructed to step as quickly as possible into four squares in a predefined sequence, stepping forward, sideways, and backward, without making contact with the canes forming the squares. The time required to complete the sequence is recorded, with shorter completion times indicating better dynamic balance. FSST completion times greater than 15 seconds are indicative of an increased risk of multiple falls (Dite & Temple, 2002). The validity and reliability of the FSST for assessing dynamic balance in older adults have been established previously (Dite & Temple, 2002).

Reactive balance was assessed with the Stepping Threshold Test (STT; Adams et al., 2021; Hezel et al., 2024) and the Dynamic stepping Threshold Test (DSTT; Hezel et al., 2023; Hezel et al., 2025).

The STT assesses static reactive balance using a perturbation treadmill that delivers unannounced anterior-posterior and medio-lateral surface translation perturbations of increasing magnitude in random order. Perturbation intensity increases across six levels à four perturbations each (one per direction left, right, forward, backward), and participants, secured by a safety harness, are instructed to use as few compensatory steps as possible. Single-step and multiple-step thresholds are defined as the perturbation level (1-6) at which one step or multiple steps (≥2) are required to regain balance for each perturbation direction. Stepping behavior can be evaluated using an all-step count evaluation (STT-ACE) and a direction-sensitive evaluation strategy (STT-DSE). Convergent and discriminant validity of the STT in older adults (Adams et al., 2021; Hezel et al., 2024), and inter-rater reliability of the evaluation strategies for classifying stepping thresholds in healthy adults and stroke patients (Handelzalts et al., 2019) have been established previously. STT-DSE scores ≤15.5 points have been suggested to discriminate between fallers and non-fallers (Hezel et al., 2024).

The DSTT, a modified version of the STT, was used to assess dynamic reactive balance (Hezel et al., 2023; Hezel et al., 2025). Participants walk on a perturbation treadmill, secured by a safety harness, at 70% of their habitual overground walking speed while receiving unannounced anterior-posterior and medio-lateral surface translation perturbations. The DSTT consists of five levels with increasing perturbation magnitudes (levels 1-5; magnitude 5-25). Each level includes eight perturbations delivered in random order and at random intervals, varying by direction (left, right, forward, backward) and swing phase (left/right). Participants are instructed to counteract the perturbations and resume normal walking as quickly as possible. For each level, a subscore is calculated as the level number multiplied by the number of successfully completed perturbations, and subscores are summed to yield a total DSTT score ranging from 0 to 120 points, with higher scores indicating better dynamic reactive balance. Preliminary convergent validity with measures of reactive, dynamic, and global balance, gait capacity, and functional mobility has been suggested previously (Hezel et al., 2025).

Both tests are predominantly terminated in the event of a fall into the harness or if excessive anxiety is reported.

**References**

Adams M, Brüll L, Lohkamp M, Schwenk M. The Stepping Threshold Test for Reactive Balance: Validation of Two Observer-Based Evaluation Strategies to Assess Stepping Behavior in Fall-Prone Older Adults. Front Sports Act Living. 2021;3:715392.

Bohannon RW, Wang YC, Gershon RC. Two-minute walk test performance by adults 18 to 85 years: normative values, reliability, and responsiveness. Arch Phys Med Rehabil. 2015;96(3):472-477.

Brooks D, Davis AM, Naglie G. The feasibility of six-minute and two-minute walk tests in in-patient geriatric rehabilitation. Can J Aging. 2007;26(2):159-162.

Brooks D, Davis AM, Naglie G. Validity of 3 physical performance measures in inpatient geriatric rehabilitation. Arch Phys Med Rehabil. 2006;87(1):105-110.

Connelly DM, Thomas BK, Cliffe SJ, Perry WM, Smith RE. Clinical utility of the 2-minute walk test for older adults living in long-term care. Physiother Can. 2009;61(2):78-87.

Delbaere K, Close JC, Mikolaizak AS, Sachdev PS, Brodaty H, Lord SR. The Falls Efficacy Scale International (FES-I). A comprehensive longitudinal validation study. Age Ageing. 2010;39(2):210-216.

Dite W, Temple VA. A clinical test of stepping and change of direction to identify multiple falling older adults. Arch Phys Med Rehabil. 2002;83(11):1566-1571.

Feeney J, Savva GM, O'Regan C, King-Kallimanis B, Cronin H, Kenny RA. Measurement error, reliability, and minimum detectable change in the mini-mental state examination, Montreal cognitive assessment, and color trails test among community-living middle-aged and older adults. J Alzheimers Dis. 2016;53:1107-1114.

Folstein MF, Folstein SE, McHugh PR. “Mini-mental state”: a practical method for grading the cognitive state of patients for the clinician. J Psychiatr Res. 1975;12(3):189-198.

Freiberger E, de Vreede P, Schoene D, et al. Performance-based physical function in older community-dwelling persons: a systematic review of instruments. Age Ageing. 2012;41(6):712-721.

Fried LP, Tangen CM, Walston J, et al. Frailty in older adults: evidence for a phenotype. J Gerontol A Biol Sci Med Sci. 2001;56(3):M146-M156.

Guralnik JM, Simonsick EM, Ferrucci L, et al. A short physical performance battery assessing lower extremity function: association with self-reported disability and prediction of mortality and nursing home admission. J Gerontol. 1994;49(2):M85-M94.

Handelzalts S, Steinberg-Henn F, Soroker N, Schwenk M, Melzer I. Inter-observer reliability and concurrent validity of reactive balance strategies after stroke. Isr Med Assoc J. 2019;21(12):773-778.

Hauer KA, Kempen GI, Schwenk M, et al. Validity and sensitivity to change of the falls efficacy scales international to assess fear of falling in older adults with and without cognitive impairment. Gerontology. 2011;57(5):462-472.

Hezel N, Buchner T, Becker C, et al. The Stepping Threshold Test for assessing reactive balance discriminates between older adult fallers and non-fallers. Front Sports Act Living. 2024;6:1462177.

Hezel N, Buchner T, Becker C, et al. Dose-response relationship of treadmill perturbation-based balance training for improving reactive balance in older adults at risk of falling: results of the FEATURE randomized controlled pilot trial. Eur Rev Aging Phys Act. 2025;22(1):8. Published 2025 May 16

Hezel N, Sloot LH, Wanner P, et al. Feasibility, effectiveness and acceptability of two perturbation-based treadmill training protocols to improve reactive balance in fall-prone older adults (FEATURE): protocol for a pilot randomised controlled trial. BMJ Open. 2023;13(9):e073135.

Kameniar K, Mackintosh S, Van Kessel G, Kumar S. The psychometric properties of the Short Physical Performance Battery to assess physical performance in older adults: a systematic review. J Geriatr Phys Ther. 2024;47(1):43-54.

Kempen GI, Yardley L, van Haastregt JC, et al. The Short FES-I: a shortened version of the falls efficacy scale-international to assess fear of falling. Age Ageing. 2008;37(1):45-50.

Lee J, Geller AI, Strasser DC. Analytical review: focus on fall screening assessments. PM R. 2013;5(7):609–621.

Lin MR, Hwang HF, Hu MH, Wu HD, Wang YW, Huang FC. Psychometric comparisons of the Timed Up and Go, one-leg stand, functional reach, and Tinetti balance measures in community-dwelling older people. J Am Geriatr Soc. 2004;52(8):1343–1348.

Marques A, Almeida S, Carvalho J, Cruz J, Oliveira A, Jácome C. Reliability, validity, and ability to identify fall status of the Balance Evaluation Systems Test, Mini-BESTest, and Brief-BESTest in older people living in the community. Arch Phys Med Rehabil. 2016;97(12):2166–2173.e1.

Montero-Odasso M, van der Velde N, Martin FC, et al. World guidelines for falls prevention and management for older adults: a global initiative. Age Ageing. 2022;51(9):afac205.

Pangman VC, Sloan J, Guse L. An examination of psychometric properties of the Mini-Mental State Examination and the standardized Mini-Mental State Examination: implications for clinical practice. Appl Nurs Res. 2000;13(4):209–213.

Pin TW. Psychometric properties of the 2-minute walk test: a systematic review. Arch Phys Med Rehabil. 2014;95(9):1759–1775.

Podsiadlo D, Richardson S. The timed “Up & Go”: a test of basic functional mobility for frail elderly persons. J Am Geriatr Soc. 1991;39(2):142–148.

Reitan RM. Trail Making Test. Tuscon, AZ, USA: Reitan Neuropsychology Laboratory; 1992.

Tombaugh TN, McIntyre NJ. The Mini-Mental State Examination: a comprehensive review. J Am Geriatr Soc. 1992;40(9):922–935.

Sánchez-Cubillo I, Periáñez JA, Adrover-Roig D, et al. Construct validity of the Trail Making Test: role of task-switching, working memory, inhibition/interference control, and visuomotor abilities. J Int Neuropsychol Soc. 2009;15(3):438-450.

Specka M, Weimar C, Stang A, et al. Trail Making Test Normative Data for the German Older Population. Arch Clin Neuropsychol. 2022;37(1):186-198.

Strauss E, Sherman EMS, Spreen O. A Compendium of Neuropsychological Tests: Administration, Norms, and Commentary. 3rd ed. Oxford University Press; 2006.

Vasunilashorn S, Coppin AK, Patel KV, et al. Use of the Short Physical Performance Battery score to predict loss of ability to walk 400 meters: analysis from the InCHIANTI study. J Gerontol A Biol Sci Med Sci. 2009;64(2):223–229.

WHO. Integrated Care for Older People (ICOPE) guideline: Locomotor Capacity – Facilitator Guide: Module 92024. Available from: <https://cdn.who.int/media/docs/default-source/mca-documents/ageing/icope-training-programme/module-9/who-icope_m9_locomotor-capacity_fg.pdf?sfvrsn=87516d88_5>.
